# Comments on: blood product transfusion in emergency department patients: a case control study of practice patterns and impact on outcome

**DOI:** 10.1186/s12245-017-0158-3

**Published:** 2017-12-06

**Authors:** Manoochehr Karami, Salman Khazaei

**Affiliations:** 1 Research Center for Health Sciences, Hamadan University of Medical Sciences, Hamadan, Iran; 2 Department of Epidemiology, School of Public Health, Hamadan University of Medical Sciences, Hamadan, Iran; 3 Department of Epidemiology and Biostatistics, School of Public Health, Tehran University of Medical Sciences, Tehran, Iran

**Keywords:** Case- control study, Matched data, Conditional logistic regression

## Abstract

Clinical decision makings according studies result require the valid and correct data collection, andanalysis. However, there are some common methodological and statistical issues which may ignore by authors. In individual matched case- control design bias arising from the unconditional analysis instead of conditional analysis. Using an unconditional logistic for matched data causes the imposition of a large number of nuisance parameters which may result in seriously biased estimates.

## Correspondence

Dear Editor:

We read with interest, the article entitled “Blood product transfusion in emergency department patients: a case-control study of practice patterns and impact on outcome” by Beyer and colleagues published in *International Journal of Emergency Medicine* [1]. While we congratulate the authors for their cogent thesis, we were concerned about a methodological issue that may have been overlooked in the peer review.

To carry out this study, the authors used a matched case-control design and control subjects were matched with cases on a one-to-one basis for many factors including ED diagnosis, hemoglobin value, age, and gender, such that individual matching for all factors was assured. However, the authors analyzed the data inappropriately using a regression model. For individual matched case-control studies, we need to use conditional logistic modeling instead of the ordinary logistic regression methodology [2].

In matched data, conditional logistic (partial likelihood) analysis provides a valid approximation of the rate ratio and adjusts for the sampling variability found in estimating standard error and confidence intervals. Using an unconditional logistic for matched data causes the imposition of a large number of nuisance parameters which may result in seriously biased estimates [2]. The take home message for readers is to use the appropriate statistical model in order to avoid analysis pitfalls that can be anticipated from the beginning.
